# Combining metal oxide affinity chromatography (MOAC) and selective mass spectrometry for robust identification of *in vivo *protein phosphorylation sites

**DOI:** 10.1186/1746-4811-1-9

**Published:** 2005-11-01

**Authors:** Florian Wolschin, Wolfram Weckwerth

**Affiliations:** 1 Max Planck Institute of Molecular Plant Physiology, 14424 Potsdam, Germany

**Keywords:** Protein phosphorylation, Nano-ESI, Seeds, IMAC, MOAC, Neutral loss, MS^3^

## Abstract

**Background:**

Protein phosphorylation is accepted as a major regulatory pathway in plants. More than 1000 protein kinases are predicted in the *Arabidopsis *proteome, however, only a few studies look systematically for *in vivo *protein phosphorylation sites. Owing to the low stoichiometry and low abundance of phosphorylated proteins, phosphorylation site identification using mass spectrometry imposes difficulties. Moreover, the often observed poor quality of mass spectra derived from phosphopeptides results frequently in uncertain database hits. Thus, several lines of evidence have to be combined for a precise phosphorylation site identification strategy.

**Results:**

Here, a strategy is presented that combines enrichment of phosphoproteins using a technique termed metaloxide affinity chromatography (MOAC) and selective ion trap mass spectrometry. The complete approach involves (i) enrichment of proteins with low phosphorylation stoichiometry out of complex mixtures using MOAC, (ii) gel separation and detection of phosphorylation using specific fluorescence staining (confirmation of enrichment), (iii) identification of phosphoprotein candidates out of the SDS-PAGE using liquid chromatography coupled to mass spectrometry, and (iv) identification of phosphorylation sites of these enriched proteins using automatic detection of H_3_PO_4 _neutral loss peaks and data-dependent MS^3^-fragmentation of the corresponding MS^2^-fragment. The utility of this approach is demonstrated by the identification of phosphorylation sites in *Arabidopsis thaliana *seed proteins. Regulatory importance of the identified sites is indicated by conservation of the detected sites in gene families such as ribosomal proteins and sterol dehydrogenases. To demonstrate further the wide applicability of MOAC, phosphoproteins were enriched from *Chlamydomonas reinhardtii *cell cultures.

**Conclusion:**

A novel phosphoprotein enrichment procedure MOAC was applied to seed proteins of *A. thaliana *and to proteins extracted from *C. reinhardtii*. Thus, the method can easily be adapted to suit the sample of interest since it is inexpensive and the components needed are widely available. Reproducibility of the approach was tested by monitoring phosphorylation sites on specific proteins from seeds and *C. reinhardtii *in duplicate experiments. The whole process is proposed as a strategy adaptable to other plant tissues providing high confidence in the identification of phosphoproteins and their corresponding phosphorylation sites.

## Background

The proteome of different developmental stages of any kind of organism reflects more directly than the genome or the transcriptome the metabolic specialisation for the actual developmental state. In plants several studies on the proteome of different developmental stages have been conducted [1]. Seed dormancy plays a crucial role in the life cycle of plants and its proteome reflects the metabolic processes during this important developmental period. However, investigation on posttranslational modifications of the proteins gives an even more detailed view on the complex nature of seed metabolism. Protein phosphorylation has been widely described as a major regulatory protein posttranslational modification influencing many important processes in living cells [2-4]. Thus, measuring protein phosphorylation is essential to reveal regulatory and signal pathways. However, the study of protein phosphorylation confronts the researcher with several hurdles.

A complicating fact is that many proteins are not only phosphorylated at one site, but on multiple sites and that each modification seems to have different regulatory functions [5,6]. Therefore, detection of protein phosphorylation and identification of phosphorylation sites are needed for the understanding of protein regulation.

Traditionally, phosphorylation is detected by specific antibodies and/or by incorporating radioactive [^32^P]orthophosphate into proteins. However, while immunolabelling is often unspecific, incorporation experiments using radioactivity might result in artificial phosphorylation events and impose waste disposal problems. Only recently has it become possible to reliably detect protein phosphorylation by resolving the proteins of interest on a gel and submitting the gel to fluorescent phosphate specific dyes followed by a staining of total protein [7-11].

The low abundance of phosphoproteins and the accurate identification of the specific phosphorylation sites, however, still impose problems.

Because of their sensitivity and resolving power, selective enrichment of phosphorylated peptides/proteins and liquid chromatography coupled to mass spectrometry (LC-MS) based methods are now widely used for phosphorylation site identification (for review see [4,12,13]).

The low abundance of phosphorylated peptides can be circumvented in part by enrichment of the phosphopeptides with IMAC (Immobilised metal affinity chromatography) after tryptic digestion of phosphoproteins. While numerous publications exist describing the enrichment of phosphopeptides from animal sources considerably less studies have been published for plant tissue (for review see [14]). These broad range studies focus for example on thylakoid proteins and plasma membrane proteins of *A. thaliana *[15,16] or on the moss *Physcomitrella patens *[17].

However, relying on just one peptide for protein identification (as is commonly done after phosphopeptide enrichment) is prone to the identification of false positives. What is more, peptides phosphorylated on serines or threonines tend to loose their phosphate group during the fragmentation process in the mass spectrometer thus further complicating correct assignment. This drawback can be circumvented by the enrichment of complete phosphoproteins since this approach leads to the identification of several peptides per protein and therefore enhances the reliability of protein identification.

Recently, we reported a novel method for the enrichment of phosphorylated proteins out of complex mixtures termed MOAC (metal oxide affinity chromatography) [18]. This method adds another tool to the repertoire of methods for the identification of phosphorylated proteins and might help to overcome some of the specificity problems associated with IMAC. In this initial study we showed that MOAC can be used to enrich phosphorylated proteins from plant leaf tissue [18].

In the present study we use MOAC for the enrichment of phosphorylated proteins from *A. thaliana *seeds and for proteins from *C. reinhardtii *cell cultures and show that the method is suitable and reproducible for different kinds of samples.

However, for the identification of the exact sites of phosphorylation further steps are necessary. As mentioned above the ionization efficiency and the quality of a phosphopeptide spectra is sometimes not good enough for reliable identification and even more difficult is the determination of the exact phosphorylation site. To circumvent these problems phosphate groups can be replaced by beta-elimination and Michael addition with more stable residues thereby increasing the ionization efficiency and improving the fragmentation behaviour [5,19-23]. Yet, these approaches sometimes result in unwanted side reactions and are difficult to perform on complex mixtures. Another promising method is the analysis of peptide sequences by electron transfer dissociation [24] but this requires sophisticated modification of the mass spectrometer not yet widely available.

The approach we employed to identify phosphorylation sites makes use of the neutral loss of H_3_PO_4 _during MS^2 ^fragmentation. The dominant neutral loss peak observed in many MS^2 ^spectra derived from serine/threonine phosphorylated peptides [25] is routinely broken down in an additional MS^3 ^step and putative phosphopeptides are automatically detected in a single LC/MS run [26]. The resulting MS^3 ^spectra are often informative enough to identify the correct peptide and the phosphorylation site in a database search. However, considering the information of both MS^2 ^and MS^3 ^data is sometimes necessary to obtain unambiguous identification [27,28].

In the present work we combine the MOAC enrichment of phosphoproteins and selective mass spectrometry for a detailed study of protein phosphorylation. Phosphorylation site identification is demonstrated for enriched seed proteins and is achieved using data-dependent MS^2 ^as well as neutral-loss triggered MS^3 ^fragmentation on a linear ion trap mass spectrometer. The proposed strategy is suitable to identify putative phosphoprotein candidates with high sequence coverage and at the same time it allows identification of corresponding protein phosphorylation sites. To avoid false positive identification of phosphorylation sites only hits with congruent MS^2 ^and MS^3 ^spectra were considered in this study. Careful validation of the data led to the identification of 16 phosphorylation sites in nine seed proteins, some of them known to be phosphorylated also in the mammalian system such as ribosomal proteins. A comparison with microarraydata showed that these proteins are mainly seed specific.

## Results

### General strategy for the identification of serine/threonine phosphorylation in plants

**Figure 1:**
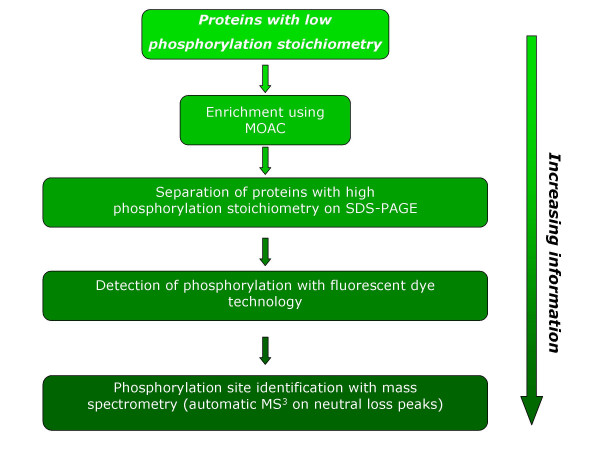
Proposed strategy for robust identification of serine/threonine phosphorylation in plants. In case of low stoichiometry as for the *in vivo *situation enrichment of phosphoproteins is necessary. To cope with this a novel enrichment procedure called metaloxide affinity chromatography (MOAC) is used in the strategy [18]. The whole approach is applicable to identify phosphorylation sites out of complex samples. The library of *in vivo *sites may be used for screening of protein phosphorylation dynamics using triple-quadruple mass spectrometry [6].

An overview on the general strategy for the identification of serine /threonine phosphorylation in plants is shown in Figure 1. The strategy includes enrichment of phosphoproteins using MOAC, phosphorylation detection using fluorescent dye technology, and determination of the phosphorylation site with neutral loss driven MS^3^. Most important, each step of the procedure gives further confidence for a robust identification of phosphoproteins and phosphorylation sites. For a detailed description see the following sections.

### MOAC enrichment of phosphoproteins

**Figure 2:**
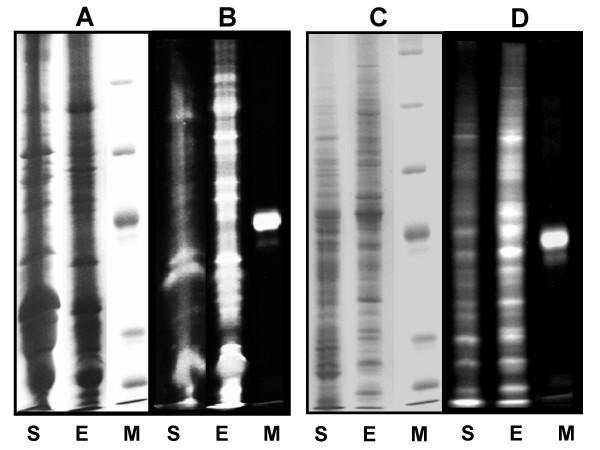
*A. thaliana *seed proteins and *C. reinhardtii *proteins before and after MOAC. M: marker S: sample (before MOAC) E: eluate (after MOAC); A: *A. thaliana *seed proteins, Coomassiestaining; B: *A. thaliana *seed proteins, Phosphostaining. C: *C. reinhardtii *proteins, Coomassiestaining; D: *C. reinhardtii *proteins, Phosphostaining. The labelled marker protein in the phosphostain is the phosphorylated protein Ovalbumin.

Proteins were extracted with phenol and enriched for phosphoproteins using metaloxide affinity chromatography (MOAC) [18]. Proteins were separated using SDS-PAGE and visualised with a phosphate-specific stain followed by staining with coomassie R-250 (Figure 1). Similar amounts of total protein from samples taken before and after MOAC (Figure 2B) are accompanied by strong differences in the phosphostain signal indicating clear enrichment of phosphoproteins out of the complex sample (Figure 2A). Bands corresponding to enriched seed proteins with a strong signal in the phosphostain were excised, digested with trypsin, and analysed by mass spectrometry (see below).

### Identification of *in vivo *phosphorylation sites in *A. thaliana *seed proteins

**Figure 3:**
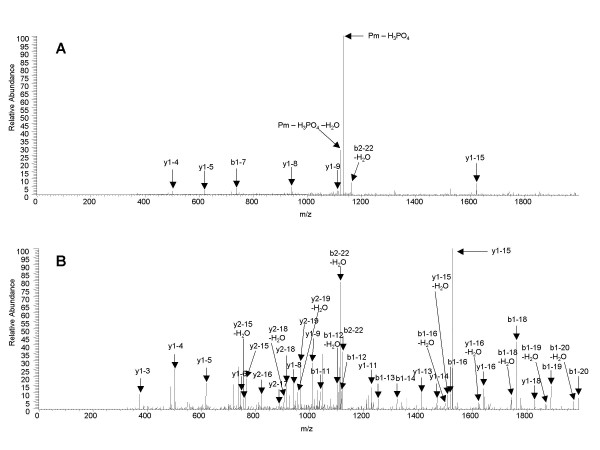
MS^2 ^spectrum and the cognate neutral loss MS^3 ^spectrum of the phosphopeptide LGYTGENGGGQSEpSPVKDETPR exemplifying the additional sequence information gained of an MS^2 ^spectrum compared to a MS^3 ^spectrum. A: MS^2 ^spectrum (Xcorr: 2.424); B: MS^3 ^spectrum (Xcorr: 3.248). Exact localisation of the phosphorylation site is only possible with the MS^3 ^spectrum in this case. Only ions above the noise signal were annotated.

Following the proposed strategy (see Figure 1) we identified after careful validation 16 phosphorylation sites in 9 proteins using the combined information of MS^2 ^and MS^3 ^data. The advantage of using the combined data is exemplified by the MS^2 ^and MS^3 ^spectra in Figure 3. In the first MS^2 ^fragmentation step a very intense fragment ion stemming from a dominant neutral loss of phosphoric acid is seen. This spectrum alone is often not suitable for database search. On the other hand it is a distinct indication for a phosphopeptide. The dominant neutral loss fragment triggers in a second scan event an MS^3 ^fragmentation step. The combination of MS^2 ^and MS^3 ^leads to the clear phosphorylation site identification with increased confidence based on the observed neutral loss of phosphoric acid [27,28]. A further level of confidence is provided by the combination of protein identification (see Figure 1 and protein sequence coverage in table 1) and the corresponding protein phosphorylation site in one LC/MS analysis. This information is missing in strategies where only phosphopeptides are enriched and protein identification is based solely on the detection of one phosphopeptide. The phosphorylation sites are shown in Table 1. The sites of the two ribosomal proteins are known to be phosphorylated in mouse [29]. For some of the proteins identified after MOAC, MS^2 ^data suggested phosphorylation but MS^3 ^data were lacking. All these ambiguous results were not included in the table. The analysis of gene expression data revealed that five of the identified proteins are apparently seed specific (table 1). Notably, phosphorylation is most probably not confunded with o-sulfonation in these experiments since sulfated peptides exhibit the characteristic loss of 80 Da instead of 98 Da during collision induced dissociation [30], which does not lead to the triggering of an MS^3 ^spectrum.

**Table 1:** Identified phosphorylation sites in seed proteins. Gene expression data were derived from a previous study [38].

Accession nr.	Description	Protein sequence coverage	Site of phosphorylation (designated as "p")	Seed specific expression
At5g52300.1	low-temperature-responsive 65 kD protein (LTI65)/desiccation-responsive protein 29B (RD29B)	65.8% (M_r _65971 Da)	MKVTDEpSPDQKSR ESDINKNpSPARFGGESK LPLSGGGpSGVKETQQGEEK LGYTGENGGGQSEpSPVKDETPR GAVTSWLGGKPKpSPR EAHQEPLNpTPVSLLSATEDVTR	+
At3g12960.1	expressed protein similar to seed maturation protein from [Glycine max]	93% (M_r _9464 Da)	DIKDIKGTRTDDpSPR.-	+
At1g01100.1	60S acidic ribosomal protein P1 (RPP1A)	81.2% (M_r _11162 Da)	KKDEPAEEpSDGDLGFGLFD.-	-
At2g27710.1	60S acidic ribosomal protein P2 (RPP2B)	87% (M_r _11444 Da)	KEEKEEpSDDDMGFSLFE.-	-
At5g40420.1	glycine-rich protein/oleosin	42.2% (M_r _21279 Da)	HFQFQpSPYEGGR	+
At1g07985.1	Expressed protein	46.5% (M_r _16461 Da)	KLVDKVVGSSSPTNIHpSK	
At5g50600.1	short-chain dehydrogenase/reductase (SDR) family protein similar to sterol-binding dehydrogenase steroleosin from [*Sesamum indicum*]	61% (M_r _39087 Da)	STLYPESIRTPEIKpSD.- ELGpSPNVVTVHADVSKPDDCRR	+
At1g29350.1	expressed protein	14.6% (M_r _90879 Da)	SGpSTHFSSTDSGNFQGK	No data
At4g25580.1	stress-responsive protein-related contains weak similarity to Low-temperature-induced 65 kDa protein	57.8 % (M_r _66520 Da)	RGAPTLTPHNTPVSLLpSATEDVTR GAPTLpTPHNTPVSLLSATEDVTR	+

### Reproducibility of the method

The enrichment process was repeated using *A. thaliana *leaf and *C. reinhardtii *proteins. Similar patterns were observed in SDS PAGE analysis combined with phospho- and coomassiestaining (data not shown). Two prominent bands, one of the enriched seed sample at about 65 kDa (corresponding to the mw of the protein with the highest number of phosphorylation sites identified in the first experiment), and one of the enriched *C. reinhardtii *sample (in duplicate) at about 56 kDa, were selected for testing the reproducibility of phosphorylation site identification. With the seed protein we tested analytical reproducibility after storage of the sample for 1 month (see table 2). Reproducibility of the whole procedure including MOAC enrichment is demonstrated by the repeated identification of phosphorylation sites in the 56 kDa *C. reinhardtii *phosphoprotein (table 2).

**Table 2:** Reproducibility test of phosphorylation site identification. +: detected; -: not detected.

Protein	At5g52300.1 low-temperature-responsive 65 kD protein		jgi code 153417 putative protein	
Mw [Da]	65971		56515	

Experiment	1	2	1	2

Sequence coverage [%]	65.8	67.5	29. 1	28.0
Peptide 1	GAVTSWLGGKPKpSPR	+	KLESAApTVAER	+
Peptide 2	LGYTGENGGGQSEpSPVKDETPR	+	VAVAPPSRPGpSGK	+
Peptide 3	LPLSGGGpSGVKETQQGEEK	+	SGpSAKVAVAPSR	-
Peptide 4	EAHQEPLNpTPVSLLSATEDVTR	+		
Peptide 5	ESDINKNpSPARFGGESK	-		
Peptide 6	MKVTDEpSPDQKSR	-		

### Phosphorylation of sterol dehydrogenase

**Figure 4:**
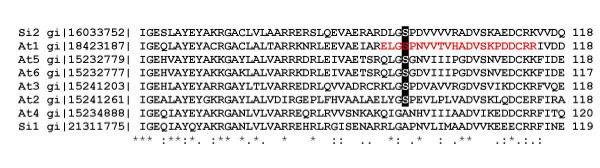
Sequence alignment of different short chain sterol dehydrogenases. Si: *Sesamum indicum*; At: *Arabidopsis thaliana*. The phosphopeptide is shown in red and the site of phosphorylation in bold.

One phosphorylation site was identified as serine 95 in an isoform of the *A. thaliana *short chain sterol dehydrogenases gene family. We aligned protein sequences belonging to the same family from *A. thaliana *(At) and *Sesamum indicum *(Si) (Figure 4). Shown is the region surrounding the phosphorylation site. The region of interest is part of a proposed NADP+ binding domain [31] and displays high homology. The serine phosphorylation site is conserved in 6 out of 8 sequences. The two remaining sequences (At4 and Si1) show no serine at the site of interest and might belong to a separate group.

## Discussion

The approach described in this work is suitable to reliably identify and routinely screen for serine/threonine phosphorylation in plant proteins. Several lines of evidence are integrated into the strategy thus making unambiguous identification of protein phosphorylation possible: (i) Enrichment for phosphoproteins based on the affinity of phosphate to MOAC, (ii) a specific phosphostain reveals phosphorylation of the proteins and confirms enrichment, (iii) gel separation of the proteins helps to guarantee high confidence in protein identification, and (iv) a highly selective method based on mass spectrometry specific for phosphorylation is used for site identification.

Phosphorylated proteins are enriched by MOAC, a method that can be easily adapted to suit the sample of interest since it is inexpensive and the components needed are widely available. For demonstration MOAC phosphoprotein enrichment was applied to *A. thaliana *leaf proteins [18], *A. thaliana *seed material and *Chlamydomonas reinhardtii *cell cultures (this study, Figure 2).

The following staining with a phosphospecific fluorescent dye is a quick and easy to use method to detect protein phosphorylation and its changes on gels. However, mass spectrometry-based site identification leads to more detailed information about site specific regulation. This holds especially true for proteins phosphorylated at multiple sites [5,6]. While it is not possible with the fluorescent stain to define if a protein is singly or multiply phosphorylated and on which amino acid the phosphate moiety is located, this can be done by mass spectrometry [32]. Consequently, enriched proteins are digested and the peptides are analysed in experiments in which an additional fragmentation event (MS^3^) is triggered when a peptide looses phosphoric acid during the first fragmentation step (MS^2^). The combined information of MS^2 ^and MS^3 ^data is then used to obtain high quality data about the peptide sequence and its phosphorylation site (see also Figure 3A and 3B).

Since this is a proof of concept study we did not aim at identifying all phosphorylation sites present in the enriched fraction but at setting up a robust and convenient workflow for the analysis of *in vivo *protein phosphorylation in plants. The major drawback of the method in its current state is that preferably phosphorylation sites of high abundant phosphoproteins are detected and that often the protein can be assigned reliably but data on the phosphopeptides are lacking. However, this might be circumvented by separating complex mixtures using established chromatography prior to MOAC and/or by coupling a phosphopeptide enrichment step to the protein enrichment. Another problem could be dephosphorylation occurring during or prior to gel separation or during sample storage. This might also explain differences in the reproducibility test. Nevertheless, most of the tested sites could be reproducibly found in a second experiment thus reconfirming the robustness of the method. However, if the sample amount is not limiting separation and digestion after enrichment might also be performed in solution without SDS-PAGE. This, of course would lead to a missing confirmation step in the strategy.

Albeit the phosphorylation of *A. thaliana *seed proteins probably plays a crucial role in seed development and dormancy [33,34], to our knowledge this is the first time a broad approach has been used to identify phosphorylation sites in seed proteins. In more than half of the identified seed derived phosphopeptides (9 out of 16) the identified phosphorylation sites are directly neighboured by a proline. Additionally, in three peptides the sites are located adjacent to aspartic acid residues. This could be due to enhanced cleavage at proline and aspartic acid residues during the fragmentation process in the mass spectrometer which has been described before [35]. Interestingly, we did find dominant neutral loss in the respective MS^2 ^spectra, even though it was reported recently that proline and aspartic acid containing phosphopeptides exhibit a less pronounced neutral loss of H_3_PO_4 _during fragmentation in a Q-Tof mass spectrometer [36]. This apparent difference might be explained by the different instrument types used in the studies or by the special nature of the investigated phosphopeptides. Both serine-proline (SP) and serine-aspartate (SD,E) containing phosphorylation sites are postulated as putative kinase substrates [15]; SP is a MAP-kinase motif which coincides with the importance of MAP-kinases in cellular processes [37] (see also ). A majority of the identified phosphoproteins appear to be seed specific since their expression is reported to be highly dominant if not exclusively expressed during this developmental stage (see table 1 and [38]).

Multisite phosphorylation seems to be quite common as indicated by the fact that more than one phosphorylation site was found in 3 of the 9 proteins. The sites identified on the two ribosomal proteins are the same c-terminal sites identified in mouse [29] thus adding another evidence for the conservation of phosphorylation sites throughout different species [39]. Phosphorylation of these ribosomal P-proteins at their c-terminal end has also been proposed for *Saccharomyces cerevisiaea*, *Rattus norvegicus*, *Trypanosoma cruzei*, and Zea mays [40]. It has been shown that phosphorylation of these proteins leads to accelerated degradation in yeast [41] and this might also hold true for *A. thaliana*. Interestingly, another two of the identified phosphopeptides, belonging to proteins otherwise unrelated to the ribosomal P-proteins were also found to be phosphorylated at their c-terminal end indicating either the accessibility of c-terminal phosphopeptides for enrichment, digestion and detection or a general pattern, putatively for protein degradation.

The identification of a phosphorylated protein with homology to a short chain sterol dehydrogenase (Sop2) seems to be especially interesting for seed development. These proteins are thought to play a vital role in plant signal transduction elicited by sterols [31,42] and therefore their phosphorylation/dephosphorylation might have large implications on seed development. Serine 95 in the peptide ELGpSPNVVTVHADVSKPDDCRR derived from Sop2 (which we showed to be phosphorylated in *A. thaliana*) is highly conserved in five out of six homologues in *A. thaliana *as well as in one out of two homologues in *Sesamum indicum *(see Figure 4). It is located in the NADP^+ ^binding domain of the protein [31] and might be important for enzyme specificity and selectivity.

## Methods

### Chemicals

All chemicals were from Sigma (München, Germany). The aluminium hydroxide was purchased as aluminium hydroxide hydrate (ordering nr. A-1577; Sigma).

### Seeds

*A. thaliana *(ecotype Columbia) seeds were taken from an in-house seed stock.

### *Chlamydomonas reinhardtii *culture

*C. reinhardtii *(wildtype CC-125) was grown for 7 days in 16/8 hour light/dark regime in liquid culture containing TAP Medium [43]. Cells were harvested in the light period and centrifuged for 10 min at 4000 rpm. The supernatant was discarded and the Pellet was used for the extraction of proteins.

### Denatured protein extraction from *A. thaliana *seeds and *C. reinhardtii*

*A. thaliana *seed proteins and *C. reinhardtii *proteins were extracted by adding a mixture of three volumes buffer-saturated phenol (15 ml) and one volume 50 mM Hepes-KOH, pH 7.2 containing 1% β-mercaptoethanol, 40 % sucrose, and 40 mM NaF (5 ml) to 2 g of seed material ground in liquid nitrogen or to a pellet derived from 100 ml of *C. reinhardtii *culture, respectively. After mixing for 20 minutes at 4°C, protein was precipitated out of the phenol phase with five volumes ice-cold acetone over night at -20°C. The pellet was washed twice with 100% ice-cold methanol and air dried for 15 min.

### Enrichment of phosphoproteins using Metal Oxide Affinity Chromatography (MOAC)

The pellet obtained by denatured protein extraction was dissolved in 1.5 ml incubation buffer containing 30 mM MES, 0.2 M potassium glutamate, 0.2 M sodium aspartate, 0.25% Chaps, and 8 M urea with a pH of 6.1. 1.5 ml of a 0.5 mg/ml protein solution was used for 30 min incubation with 80 mg of AlOH_3 _at 4°C (AlOH_3 _was washed before once with the incubation buffer described above). The incubation was followed by five washing steps of 1.6 ml and one step of 0.8 ml. Finally, proteins were eluted from the matrix by incubation with 800 μl of 100 mM potassium pyrophosphate buffer pH 9.0 containing 8 M urea for 30 min at RT. Proteins were precipitated with methanol/chloroform prior to gel loading as described by Wessel & Fluegge [44] and about one half of the eluted protein fraction was loaded onto the gel.

### Determination of protein content

Protein concentrations were determined via the dye-binding method of Bradford as described previously [45], using ovalbumin as a standard. Each measurement was made in triplicate and the mean values were used.

### SDS-PAGE, Phosphostaining, and Coomassie staining

Pellets derived from the methanol/chloroform precipitation were dissolved in 2 × SDS sample buffer (90 mM Tris, pH 6.9, 20% Glycerin, 2% SDS, 0.02% bromophenol-blue, and 100 mM DTT) and approximately 30 μg were subjected to SDS-PAGE (800 μl MOAC eluate fraction was precipitated and dissolved in 40 μl of 1 × SDS sample buffer). After the separation proteins were stained with Pro-Q diamond stain (Invitrogen, Karlsruhe, Germany) essentially following the instructions in the manual. For specificity, we found exchanging fixation solution once (after 30 min) and leaving the gels in the fixation solution over night to be crucial steps. Phosphorylation was visualised using a chemdoc station and a 550 nm filter. After visualisation the phosphostain gels were washed three times with ddH_2_O and stained with coomassie.

### In-gel tryptic digest

Seed protein spots exhibiting a strong signal after phosphostaining were excised and digested over night with trypsin as described before [46]. Peptides were extracted from the gel in three consecutive steps using increasing percentages of acetonitrile (5 %, 50 %, 90% each containing 1% formic acid).

### Protein and phosphorylation site identification using nano LC-linear-iontrap-MS

Peptides were loaded directly onto a ProteoSpher^® ^Micro column (0.5 mm × 15 mm) at a flow rate of 3 μl/min and separated in a 85 min gradient from 80% A (0.1% formic acid, and 2.5 % acetonitrile in water) to 100 % B (0.1% formic acid in methanol). Separation and measurements were performed with a nano-LC-pump (Agilent 1100) coupled to an LTQ ion trap (Thermoelectron) with a nano-ESI-source. The voltage was applied directly to the analyte solution using a T-piece. To identify tryptic peptides, phosphopeptides and phosphorylation sites, automatic data-dependent acquisition was performed consisting of a full scan (m/z 400–2000), a subsequent MS^2^, and a neutral loss scan of 98, 49, or 32.7 (H_3_PO_4 _for the +1, +2, and +3 charged ions, respectively) in the five most abundant MS^2 ^fragments. An MS^3 ^scan was automatically collected on the corresponding neutral loss fragments of the MS^2 ^scan events. Peptides were identified by searching the spectra against an *A. thaliana *database  or against the *C. Chlamydomonas *database version 2.0  containing trypsin and keratin sequences using the Sequest algorithm (ThermoElectron, Dreieich, Germany) and filtering the results with an Xcorr of 2.0, 2.5, and 3.5 for singly, doubly, and triply charged ions, respectively. Protein hits were accepted when at least three peptides with the corresponding Xcorr criteria described above were identified. The spectra derived from phosphopeptides were verified manually (charge state and identification of MS^2 ^and MS^3 ^spectra were checked for their concordance).

### Sequence alignment

Sequences were derived from NCBI  and alignment was performed using ClustalW  with the default settings.

## Authors' contributions

FW carried out the optimization of MOAC with seed material, phosphorylation site identification using MS^2 ^and MS^3 ^and participated in writing and drafting the manuscript. WW drafted the conception of this study, advised throughout the project and participated in writing and drafting the manuscript.
